# Effects of Al/Na and Si/Na Molar Ratios on the Alkalinity of Metakaolin-Based Geopolymer Pore Solutions

**DOI:** 10.3390/ma16051929

**Published:** 2023-02-25

**Authors:** Weiwei Han, Yigang Lv, Shiyu Wang, Jie Qiao, Chaosheng Zou, Miao Su, Hui Peng

**Affiliations:** 1 National Engineering Research Center of Highway Maintenance Technology, Changsha University of Science & Technology, Changsha 410114, China; 2 School of Traffic & Transportation Engineering, Changsha University of Science & Technology, Changsha 410114, China; 3 School of Civil Engineering, Changsha University of Science & Technology, Changsha 410114, China; 4 Guangdong Hualu Communications Technology Co., Ltd., Guangzhou 510550, China; 5 Zhejiang Communications Construction Group Co., Ltd., Hangzhou 310051, China

**Keywords:** metakaolin, geopolymer, molar ratio, alkalinity, microstructure

## Abstract

The pH values of pore solutions are of great significance for the durability of concrete, but the influencing factors and mechanisms of geopolymer pore solutions are still unclear and the composition of raw material elements has a great influence on the geological polymerization behavior of geopolymers. Therefore, we prepared geopolymers with different Al/Na and Si/Na molar ratios using metakaolin, and the pH and compressive strength values of the pore solutions were determined using solid–liquid extraction. Finally, the influencing mechanisms of sodium silica on the alkalinity and geological polymerization behavior of geopolymer pore solutions were also analyzed. The results showed that the pH values of the pore solutions decreased with an increase in the Al/Na ratio and increased with an increase in the Si/Na ratio. The compressive strength of the geopolymers first increased and then decreased with an increase in the Al/Na ratio and decreased with an increase in the Si/Na ratio. The exothermic rates of the geopolymers first increased and then slowed down with an increase in the Al/Na ratio, indicating that the reaction levels first increased and then decreased with an increase in the Al/Na ratio. The exothermic rates of the geopolymers gradually slowed down with an increase in the Si/Na ratio, indicating that an increase in the Si/Na ratio reduced the reaction levels. In addition, the results obtained from SEM, MIP, XRD and other test methods were consistent with the pH change laws of geopolymer pore solutions, i.e., the higher the reaction level, the denser the microstructure and the smaller the porosity, whereas the larger the pore size, the smaller the pH value of the pore solution.

## 1. Introduction

Geopolymers are inorganic cementitious materials with zeolite-like architectures or spatial network structures that are mainly composed of ionic bonds and covalent bonds. The main raw materials of geopolymers are aluminosilicate natural ores (e.g., metakaolin) or industrial waste (e.g., fly ash and slag), and they are generated via excitation (mainly alkali activators) [1,2]. As a new type of low-carbon green material, the preparation processes of geopolymers produce much fewer carbon emissions than those of Portland cement. Moreover, geopolymers have excellent properties, such as fast hardening and early strengthening, which make it an ideal substitute for traditional cementitious materials [3,4,5]. The performance of geopolymers that are made from slag and fly ash is greatly affected by the place of origin and manufacture processes of the raw materials. In addition, fly ash-based geopolymers need to be cured at high temperatures to increase their strength, while the compressive strength of slag-based geopolymers decreases significantly in the later stages of production [6,7,8]. In contrast, metakaolin is rich in silicon–aluminum oxides and has high volcanic ash activity [9]. Geopolymers that are made from metakaolin usually have high compressive strength and stable performance [10].

As a new type of material, geopolymers have been extensively studied by many scholars. However, the current research on geopolymers has only focused on the selection of raw materials, as well as their activators, maintenance rules and macroscopic mechanical properties [11,12,13,14,15]. There have only been a few studies on the pH (Pondus Hydrogenii) values of pore solutions. The pH of pore solutions is an important factor that affects the carbonization of concrete and the corrosion of steel. Identifying the appropriate pH values for pore solutions is also of great significance for the stability of the hydration products of cementitious materials [16,17]. Portland cement contains a large amount of CaO, which generates Ca(OH)_2_ in water. Thus, the pore solution of Portland cement concrete maintains a high alkalinity level. This high alkalinity environment is beneficial for the generation of complete and dense passivation films on the surface of steel bars, which protects the steel bars from corrosion [18]. In contrast, geopolymers are mainly excited by alkali solutions and their pore solutions have higher initial alkalinity than that of Portland cement. There is almost no Ca(OH)_2_ in metakaolin-based geopolymers, so it is impossible to maintain the supplementary alkalinity through the continuous dissolution of Ca(OH)_2_ [19]. Hence, this geopolymer slurry is more susceptible to carbonization and ion erosion. Compared to Portland cement concrete, geopolymer concrete is also easier to carbonize [20,21]. Carbonization causes a decrease in the pH values of pore solutions, which causes the corrosion of internal steel bars [18]. This seriously restricts the application of geopolymer concrete in practical engineering. Therefore, the initial pH values and alkalinity of metakaolin-based geopolymers are important issues in the study of the application of geopolymer concrete.

Currently, studies regarding the pH values and influencing factors of geopolymer pore solutions have been limited [22]. Shi et al. [23] found that the greater the concentration of the alkali activator, the higher the pH of the geopolymer pore solution. Unlike Portland cement concrete, geopolymers are produced by alkali activators. Although the pore solutions contain a large number of OH^−^ ions and have higher initial pH values, they do not contain any calcium hydroxide, which makes it is difficult to maintain their alkalinity. Lv et al. [24] found that the true pH of the porous solution of a metakaolin-based geopolymer exceeded 12.75 and that a decrease in the modulus of the excitation agent increased the pH of the geopolymer pore solution. Experiments by Pouhet et al. [21] showed that metakaolin-based geopolymers were almost completely carbonized within one year and that the value pH decreased from 14 to 10.5. Therefore, although the risk of rebar corrosion at this pH is not significant, the negative effects of this change in the alkalinity of pore solutions on the passivation of steel bars need to be considered. In addition, Wang et al. [25] analyzed the elemental compositions of raw materials and found that the Si/Al and Na/Al molar ratios played important roles in geological polymerization behavior and the material properties of metakaolin-based polymers, which offered a relatively novel perspective for the study of the properties of geopolymers.

The study of the pH values of pore solutions is important for our understanding of geopolymer carbonization, steel corrosion and slurry stability. Compared to Portland cement, the pore solutions of metakaolin-based geopolymers have higher initial alkalinity, but the alkali distribution ranges, influencing factors and mechanisms that are not clear. In addition, the change laws of the alkalinity of pore solutions with the increase in age are not well understood. In light of this, in this work, we analyzed a range of metakaolin-based geopolymers, and the Al/Na and Si/Na molar ratios were used as experimental variables. The pH and compressive strength values of the pore solutions at different curing ages were tested using solid–liquid extraction. The influence of the Al/Na and Si/Na molar ratios on the pH of the pore solutions and the change mechanisms of the pH of the pore solutions were then explored in detail.

## 2. Experimental Setup

### 2.1. Raw Materials

The metakaolin used in this study was the K-1300-type metakaolin with high activity, which was provided by the Inner Mongolia Chaopai Building Materials Technology Co., Ltd. The average particle size and specific surface area of the metakaolin were 3 μm and 15 cm^2^/g, respectively. The alkali activator was made of modified sodium silicate solution (SiO_2_/Na_2_O molar ratio = 3.28; solid content = 34.89%). The other ingredients included industrial-grade flake sodium hydroxide (with a purity of 99.5%) and deionized water.

The chemical composition of the metakaolin was determined using X-ray fluorescence spectroscopy (XRF) and the results are shown in Table 1.

**Figure 1:**
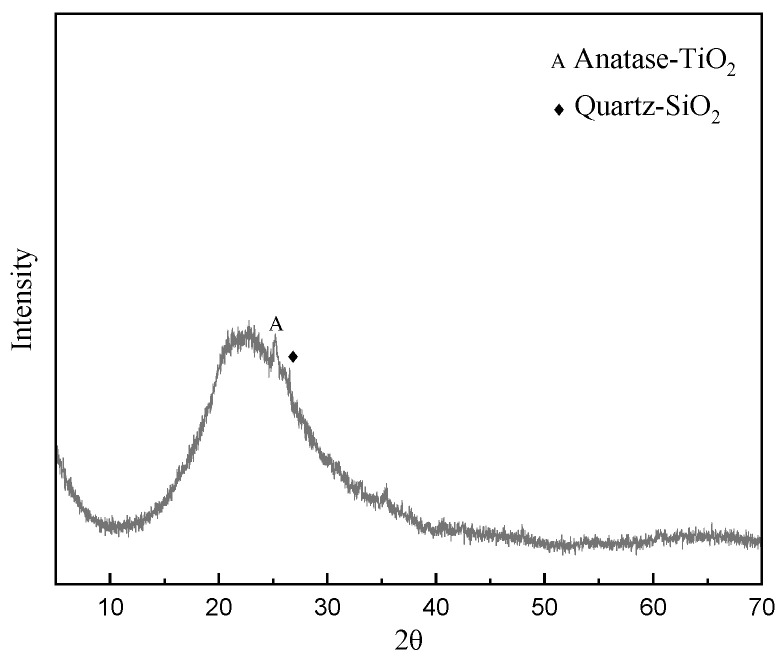
The XRD diagram of the K-1300 metakaolin that was used in this study.

Figure 1 shows the phase composition of the metakaolin, which was analyzed using an X-ray diffractometer (XRD). The main diffraction peak of the metakaolin was between 2θ = 15°–30° and the main crystal phases were anatase (TiO_2_) and quartz (SiO_2_).

### 2.2. Mix Design

According to existing research [26,27,28], the silicon, aluminum and sodium contents are important influencing factors for the synthesis of geopolymers. Therefore, considering the flow performance during the preparation of the geopolymer specimens [29,30,31], the Al/Na and Si/Na ratios were taken as the variables for the metakaolin-based geopolymer specimens, as shown in Table 2. The specimens were cured under the standard conditions of a temperature of 20 °C and a humidity of 90%. Then, the effects of the variables on the pH of the pore solutions of the geopolymer specimens at different ages (i.e., 1 d, 3 d, 7 d, 14 d and 28 d) were studied.

Before preparing the geopolymers, the initiator needed to be formulated to the necessary requirements for the mix ratio. The two main parameters of the sodium silicate liquid were the modulus and concentration: the Na_2_O content in the initiator was adjusted by adding solid sodium hydroxide during the preparation process and the concentration of the initiator was adjusted by adding deionized water. In this experiment, we assumed that the amount of raw metakaolin material in the whole system was 100 g and then we calculated the SiO_2_ and Al_2_O_3_ molar numbers in the raw metakaolin material. Since Al_2_O_3_ was completely provided by the raw metakaolin material, the number of Al_2_O_3_, Na_2_O and SiO_2_ moles in the whole system and the amount of deionized water could be calculated according to the Al/Na ratio, Si/Na ratio and excipient concentration. According to the number of Al_2_O_3_ and SiO_2_ moles, the number of SiO_2_ moles required in the initiator (the number of SiO_2_ moles in the whole system minus the number of SiO_2_ moles in the raw metakaolin) and the amount of raw sodium silicate could be calculated. The amount of NaOH could be calculated according to the amount of commercially available sodium silicate and the number of Na_2_O moles in the whole system (calculated according to the number of Na_2_O moles in the whole system minus the number of Na_2_O moles in the original sodium silicate). Finally, the initiator could be adjusted to the pre-set test values. The specific mix ratio designs are shown in Table 2.

### 2.3. Specimen Manufacture

After aging the prepared alkali initiator solution for 24 h, the amount of metakaolin was weighed according to the mix ratio and pre-mixed in a blender. After the metakaolin particles were evenly dispersed without bonding, the alkali activator solution was added to the metakaolin. The obtained slurry was first stirred at a low speed for 2 min and then at a high speed for 3 min. Subsequently, the stirred slurry was injected into a cylindrical mold with a diameter of 50 mm and a height of 50 mm. The slurry was vibrated on a shaker table for 3 min to remove any bubbles. Lastly, the slurry was put into a standard curing box for 24 h. After demolding, the specimens were kept in a standard curing environment until they reached the specified age.

### 2.4. Test and Analysis Methods

#### 2.4.1. Pore Solution Test

At present, the commonly used pH test methods for pore solutions include the high-pressure pressing method and the solid–liquid extraction method [32,33]. For the high-pressure pressing method, specimens are placed in a filter press device and the solution in the internal pores of the specimens is squeezed out by a high-pressure stress of 500~600 MPa. Then, the pH of the pore solution is tested. This method is highly accurate but also has a lot of requirements, complex operation and some other limitations [34,35,36]. The solid–liquid extraction method crushes and grinds the specimens and then passes them through a standard sieve with a pore size of 75 μm. The obtained powder is soaked and dried with absolute ethanol. The dried powder is mixed with deionized water at a mass ratio of 1:10 and then the pH of the supernatant can be tested. The solid–liquid extraction method for determining the alkalinity of pore solutions is quick and simple to operate and has good applicability. Moreover, any changes in the pH of pore solutions can be characterized by the amount of exudate OH^−^ in the pores [37,38]. In light of this, the solid–liquid extraction method was selected to test the pH of pore solutions in this study.

In order to prevent carbonization, (3 ± 0.0003) g of drying powder and 30 mL of deionized water were added into a sealed 50 mL plastic bottle. The mixture was sealed and stirred for 30 min and then rested for 2 h. Then, 10 mL of supernatant was poured into a centrifuge tube for 5 min at a rate of 4000 r/min. Taking 5 mL of clear liquid, the amount of OH substance in the solution was measured using a Metrohm 848 Titrino Plus automatic potentiometric titrator. Each group of tests was repeated three times and the average value was used. The pH was calculated as follows:

(1) $$\mathrm{pH}=14+\mathrm{lg}\frac{a\left({\mathrm{OH}}^{-}\right)}{5}$$

where a is the amount of OH^−^ matter and the unit is mol.

#### 2.4.2. Microstructure Test

The micromorphology of the geopolymer specimens was also observed using an SEM (scanning electron microscope). The specimens were crushed after 28 days of curing. Then, any fragments with flat surfaces that were less than 1 cm^3^ were soaked in absolute ethanol for 48 h and dried to a constant weight at 50 °C. A ZEISS-EVO-MA-25 series high-resolution SEM was used in this test. The flat cross-sections of the fragments were gold sprayed to observe the microscopic topographies of the samples.

In addition, the MIP (mercury intrusion porosimetry) method was used to test the pore structures of the geopolymer specimens. A PoreMaster 33 series automatic pore size analyzer was used to determine the porosity and pore size distributions of the test samples.

#### 2.4.3. Phase Composition Analysis

The phase compositions of the geopolymer specimens were analyzed using the XRD (X-ray diffractometer) method. A standard 200 mesh sieve was used for the powder tests. An Ultima-IV series XRD was used, the scanning range and speed of which were 5–70° and 4°/min, respectively.

## 3. Results and Discussion

### 3.1. The pH Analysis of the Pore Solutions

#### 3.1.1. Effects of the Al/Na Ratio

**Figure 2:**
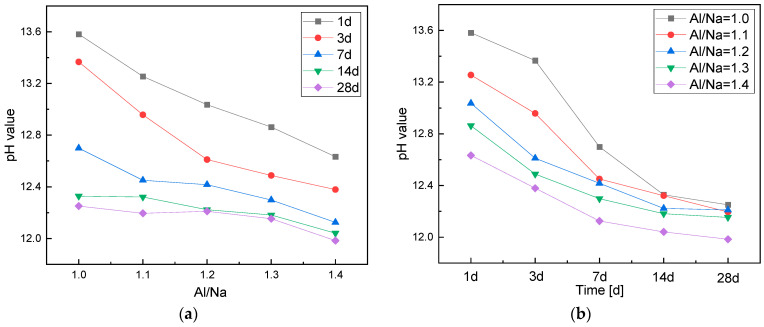
(**a**) A chart of the changes in the pH of the pore solutions with Al/Na ratio; (**b**) a chart of the changes in the pH of the pore solutions with age.

The changes in pH values with Al/Na ratio and the age of the specimens are shown in Figure 2. As can be seen from Figure 2a, the pH values decreased as the Al/Na ratio increased. Since the Al component in the raw material gradually increased, more OH^−^ needed to be consumed in the early stages of geopolymerization to break the Al–O bonds and form AlO_4_ tetrahedrons. As a result, the amount of alkali remaining decreased and the pH values of the pore solutions continuously decreased. In addition, when there was too much Al in the raw material, the reaction levels largely depended on the amount of alkali in the activator. The Na^+^ in the solutions gradually decreased, which caused a decrease in alkalinity. Therefore, the metakaolin particles could not be further dissolved and some OH^−^ remained in the pore solutions to maintain their alkalinity. Then, the pH of the geopolymer pore solutions with different Al/Na ratios tended to eventually become stable.

As illustrated in Figure 2b, in the early stages of geopolymerization, the pH of each specimen decreased with the increase in age. The pH basically tended to become stable after 14 days. The geopolymerization rate was fast in the early stages, consuming a large amount of OH^−^, which caused the pH to drop rapidly. On the other hand, with the increase in age, geopolymerization was almost completed. Then, the OH^−^ in the pore solutions was redistributed, so any changes in the pH values were small. At this time, the reaction rate was mainly controlled by the ion diffusion rate. As the OH^−^ content of the pore solutions decreased, the rate of geopolymerization decreased. The surface of the remaining unreacted particles was covered by the generated N-A-S-H gel, which made it difficult for the OH^−^ to break through the gel layer and enter the particle to react. Hence, the OH^−^ content in the pore solutions only changed a little, resulting in a slower rate of change in the pH.

#### 3.1.2. Effects of the Si/Na Ratio

**Figure 3:**
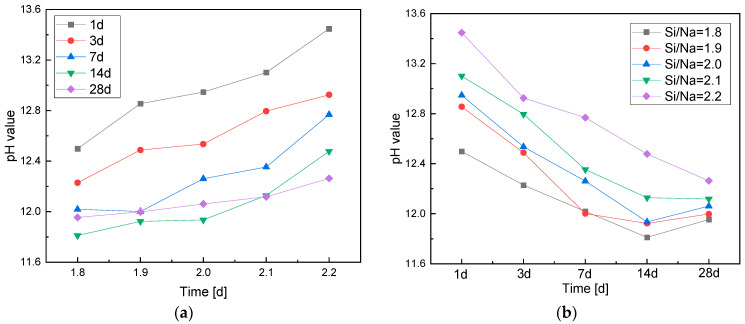
(**a**) A chart of the changes in the pH of the pore solutions with Si/Na ratio; (**b**) a chart of the changes in the pH of the pore solutions with age.

The change laws of the pH values of the pore solutions with Si/Na ratio and age are shown in Figure 3. The pH increased as the Si/Na ratio increased. When the Si/Na ratio was relatively small, the systems contained a large amount of Na^+^. At this time, the alkali activator was strongly alkaline, which promoted geopolymerization. The dissolved Al component promoted the dissolution of metakaolin to generate more N-A-S-H gel. Therefore, the reaction levels of the geopolymers were high, resulting in the low pH values of the pore solutions as the OH^−^ consumption was large. With the increase in Si/Na ratio, the amount of raw metakaolin material decreased. Therefore, the aluminum contents in the whole systems were insufficient and the reaction levels of the geopolymers became low. Therefore, OH^−^ consumption was reduced and the pH of the pore solutions increased.

It can be seen from Figure 3b that with the increase in the curing age of the geopolymers, the pH of the pore solutions initially decreased. The pH values were the lowest at the age of 14 days. Then, the PH increased slightly and tended to become stable. With the increase in Si/Na ratio, the pH of the pore solutions became more obviously affected by the curing age. The geopolymerization rate was relatively fast as a high reaction level could be reached in a short amount of time. However, with the increase in curing age, the internal free alkali of the geopolymers and the unreacted raw metakaolin material were further dissolved and polymerized. During this process, some of the alkali was consumed, which eventually led to a decrease in the pH of the geopolymer pore solutions. When the reaction was finished, the microstructures changed and the OH^−^ was redistributed, so the pH of the pore solutions increased slightly. After 14 days, the microstructures and pH values of the pore solutions were basically stable. The larger the Si/Na ratio, the weaker the alkalinity of the alkali activator. Thus, the slower the early reaction rate of the specimen, the lower the dissolution rate of the Al component. The dissolved Al component promoted the dissolution of metakaolin, which accelerated geopolymerization. The reaction rates of the systems became faster with the increase in age and the pH of the pore solutions changed significantly with the increase in the consumption of OH^−^. Hence, the pH of the pore solutions of specimens with larger Si/Na ratios was more greatly affected by age.

### 3.2. Analysis of Compressive Strength

#### 3.2.1. Effects of the Al/Na Ratio

**Figure 4:**
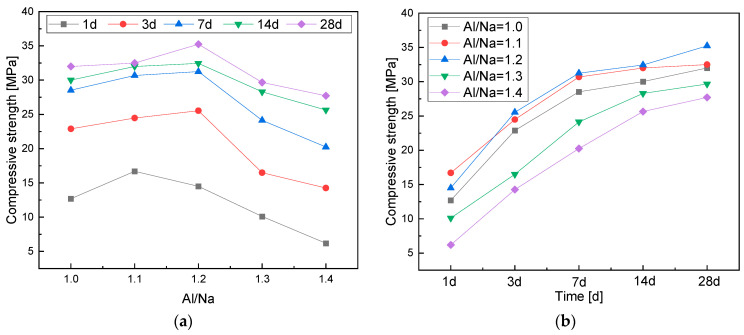
(**a**) A chart of the changes in compressive strength with Al/Na ratio; (**b**) a chart of the changes in compressive strength with age.

The change laws of the compressive strength of the metakaolin-based geopolymers with Al/Na ratio and age are shown in Figure 4. As shown in Figure 4a, the compressive strength first increased and then decreased with the increase in Al/Na ratio over the same curing period. The Al components in the systems gradually increased with the increase in Al/Na ratio, which promoted geopolymerization, made the structures of the geopolymers become denser and generated higher compressive strength [30]. As the Al/Na ratio continuously increased, the alkali contents in the systems gradually decreased, resulting in an excess of Al components. At this time, geopolymerization was mainly affected by the alkali content in the system. Therefore, the increase in Al/Na ratio reduced the reaction rates and reaction levels, leading to a decrease in the compressive strength of the geopolymers.

It can be seen from Figure 4b that as the curing age increased, the geological polymerization reactions continued and more N-A-S-H gels were produced in the system. Wang et al. [25] found that the macroscopic strength of geopolymers mainly depended on the formation of N-A-S-H gels and that more N-A-S-H gels gradually densified the structures of metakaolin-based polymers, which improved the compressive strength of the geopolymers. In the early stages of the reactions, the Al components in the systems that had higher activity increased the early reaction rates of the geopolymers. Therefore, the growth rate of compressive strength during the early stages of curing was fast. There was also an excess of Al components in the raw material during the subsequent reactions. The reaction rates mainly depended on the amount of alkali in the activator. However, the alkali was largely consumed during the subsequent reactions and the hydration reaction rates decreased. Thus, the compressive strength of the metakaolin-based geopolymers gradually stabilized during the late stages of curing.

#### 3.2.2. Effects of the Si/Na Ratio

**Figure 5:**
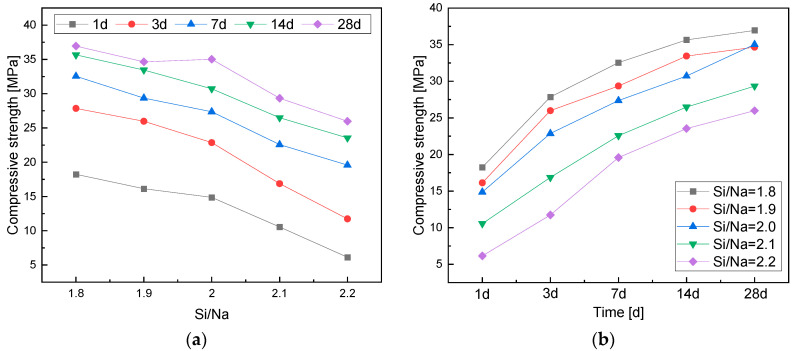
(**a**) A chart of the changes in compressive strength with Si/Na ratio; (**b**) a chart of the changes in compressive strength with age.

It can be seen from Figure 5a that the compressive strength of the geopolymers gradually decreased with the increase in Si/Na ratio over the same curing age. The alkali contents in the geopolymer systems decreased with the increase in Si/Na ratio. Meanwhile, the amount of Al components in the systems that had higher activity decreased, resulting in slower reaction rates and lower reaction levels. A large amount of metakaolin did not react. Hence, the compressive strength of the geopolymers gradually decreased [30].

The compressive strength of the geopolymers initially increased rapidly when the Si/Na ratio was small (Figure 5b) because the alkali contents in the systems and the hydration reaction rates were high. Wan et al. [27] found that when the Si/Na ratio was relatively large, high concentrations of Si and Al dissolved from the raw material to form high-content Si–O–T bonds and that the dissolved Al components in the systems increased the reaction rates and reaction levels of geological polymerization to a certain extent. Therefore, the compressive strength of the metakaolin-based polymers gradually increased and tended to finally become stable with the increase in curing age.

### 3.3. Geopolymerization

#### 3.3.1. Study of Reaction Levels under Changes in Al/Na Ratio

**Figure 6:**
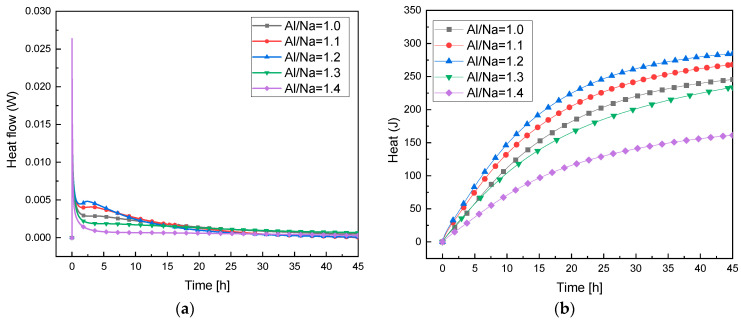
(**a**) The exothermic reaction rates at different Al/Na ratios; (**b**) the total exothermic reaction map at different Al/Na ratios.

Figure 6 shows a comparison of the exothermic reaction rates of the raw materials under different Al/Na ratios. The exothermic rates first increased and then gradually slowed down with the increase in Al/Na ratio, indicating that the reaction levels rapidly increased with the increase in Al/Na ratio. The relative consumption of metakaolin increased with the increase in Al/Na ratio. The Al_2_O_3_ in the raw soil was dissolved in the alkaline environment. As a result, more OH^−^ ions were consumed and more [Al(OH)_4_]^−^ was generated. Meanwhile, SiO_2_ was dissolved and generated [SiO_4_]^4−^. Na^+^ ions shuttled through the interlayer equilibrium points of the structures to generate N-A-S-H gels, which increased the reaction levels. However, the continuous increase in Al/Na ratio reduced the number of Na^+^ and OH^−^ ions. Although the activity of Al was higher, there were not enough OH^−^ ions to dissolve the active Al in metakaolin and not enough Na^+^ ions to balance the negative charge of the [Al(OH)_4_]^−^. Thus, more gels could not be generated. Hence, the reaction levels continued to decrease with the increase in Al/Na ratio. It can be seen from Figure 6b that the cumulative exothermic reaction also first increased and then tended to gradually become stable with the increase in Al/Na ratio, indicating that the reaction levels first increased and then decreased.

#### 3.3.2. Study of Reaction Levels under Changes in Si/Na Ratio

**Figure 7:**
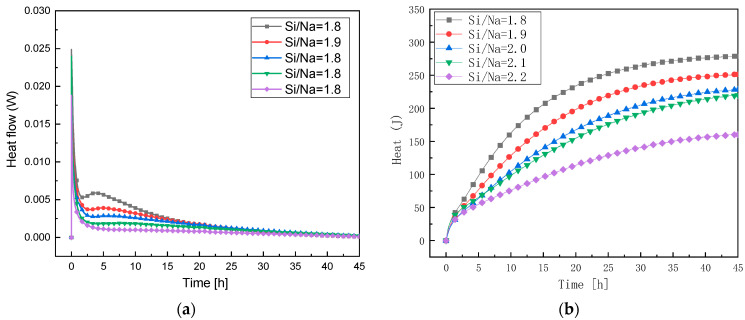
(**a**) The exothermic reaction rates at different Si/Na ratios; (**b**) the total exothermic reaction map at different Si/Na ratios.

It can be seen from Figure 7a that with the increase in Si/Na, the exothermic reaction rates gradually slowed down, indicating that the increase in Si/Na ratio decreased the reaction rates. The smaller the Si/Na ratio in the raw material, the higher the alkali content in the system. Therefore, the concentrations of OH^−^ ions and Na^+^ ions in the activator increased. The increase in OH^−^ concentration accelerated the dissolution of active Al in the metakaolin and the increase in Na^+^ concentration promoted the polymerization of [Al(OH)4]^−^ into N-A-S-H gel. As a result, lower Si/Na ratios increased the response levels of the metakaolin. As shown in Figure 7b, the cumulative exothermic reaction gradually decreased with the increase in Si/Na ratio, indicating that the increase in Si/Na ratio reduced the reaction levels. This conclusion was consistent with the results of the exothermic reaction rates.

### 3.4. Microstructure Analysis

#### 3.4.1. SEM Diagrams and Pore Structures with Different Al/Na Ratios

**Figure 8:**
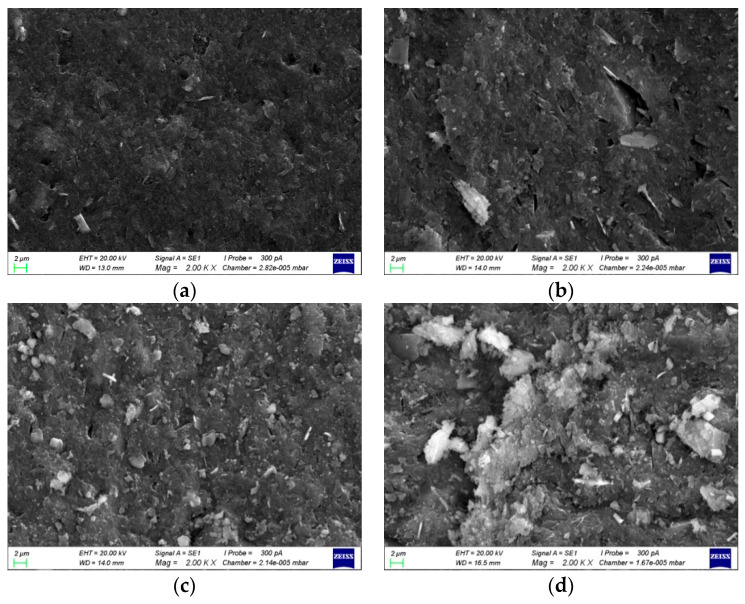

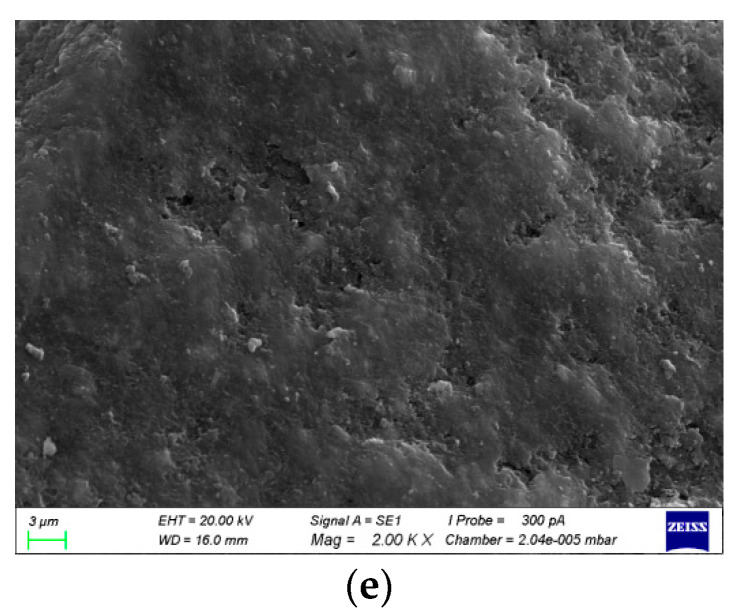
(**a**) The Al/Na ratio was 1.0 and the specimen was amplified 2000 times; (**b**) the Al/Na ratio was 1.1 and the specimen was amplified 2000 times; (**c**) the Al/Na ratio was 1.2 and the specimen was amplified 2000 times; (**d**) the Al/Na ratio was 1.3 and the specimen was amplified 2000 times; (**e**) the Al/Na ratio was 1.4 and the specimen was amplified 2000 times.

The microscopic morphologies of the sample surfaces with different Al/Na ratios after 28 days of curing are shown in Figure 8. The microstructures became denser with the increase in Al/Na ratio. The degree of reaction gradually increased and more OH^−^ was consumed in the specimens. Therefore, the pH of the geopolymer pore solutions continuously decreased with the increase in Al/Na ratio, which was consistent with the aforementioned conclusion.

**Figure 9:**
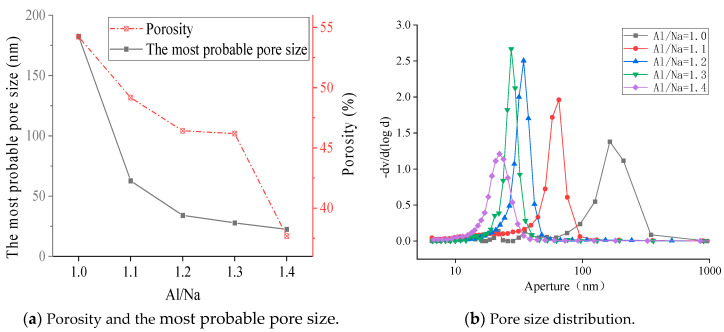
Changes in the pore structure parameters of the geopolymer samples with different Al/Na ratios.

The MIP (mercury intrusion porosimetry) method was used to test the pore structures of the geopolymer specimens. The porosity, most probable pore size and pore size distribution for different Al/Na ratios are shown in Figure 9. The porosity and most probable pore size when Al/Na = 1.0 were 54.22% and 182.64 nm, respectively. Both the porosity and the most probable pore size were relatively large. When the Al/Na ratio increased to 1.4, the porosity and most probable pore size decreased to 37.67% and 22.38 nm, respectively, which significantly improved the pore structure. The increase in Al/Na ratio increased the degree of reaction. Moreover, the contents of the formed gels greatly increased and the pore structures were filled, so the pore size gradually became refined and the porosity and most probable pore size reduced. Meanwhile, the increase in reaction degree also showed that more OH^−^ was consumed during the geopolymerization process. The pH values of the measured pore solutions gradually decreased with the increase in Al/Na ratio, indicating that the consumption of OH^−^ increased. This was consistent with the pore structure analysis results.

#### 3.4.2. SEM Diagrams and Pore Structures with Different Si/Na Ratios

**Figure 10:**
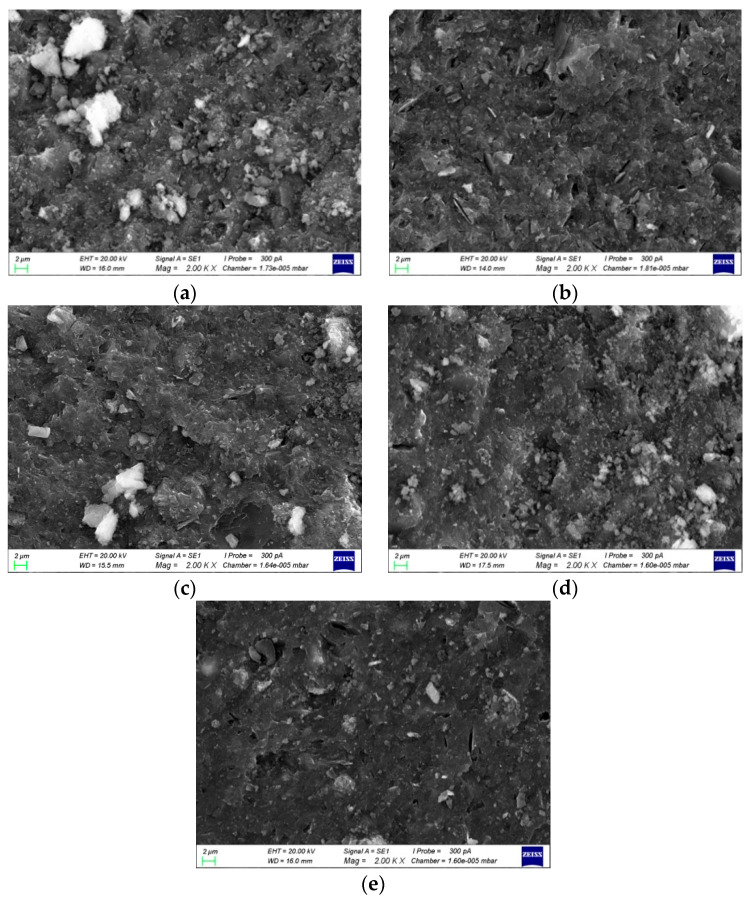
(**a**) The Si/Na ratio was 1.8 and the specimen was amplified 2000 times; (**b**) the Si/Na ratio was 1.9 and the specimen was amplified 2000 times; (**c**) the Si/Na ratio was 2.0 and the specimen was amplified 2000 times; (**d**) the Si/Na ratio was 2.1 and the specimen was amplified 2000 times; (**e**) the Si/Na ratio was 2.2 and the specimen was amplified 2000 times.

The microscopic morphologies of the sample surfaces with different Si/Na ratios after 28 days of curing are shown in Figure 10. It can be seen from the figure that as the Si/Na ratio increased, the microstructures became looser. This showed that the degree of reaction gradually decreased and the remaining OH^−^ in the structures increased, which increased the pH values of the pore solutions.

**Figure 11:**
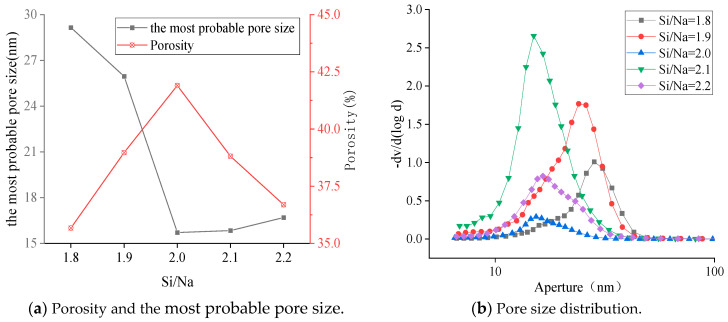
Changes in the pore structure parameters of the geopolymer samples with different Si/Na ratios.

**Figure 12:**
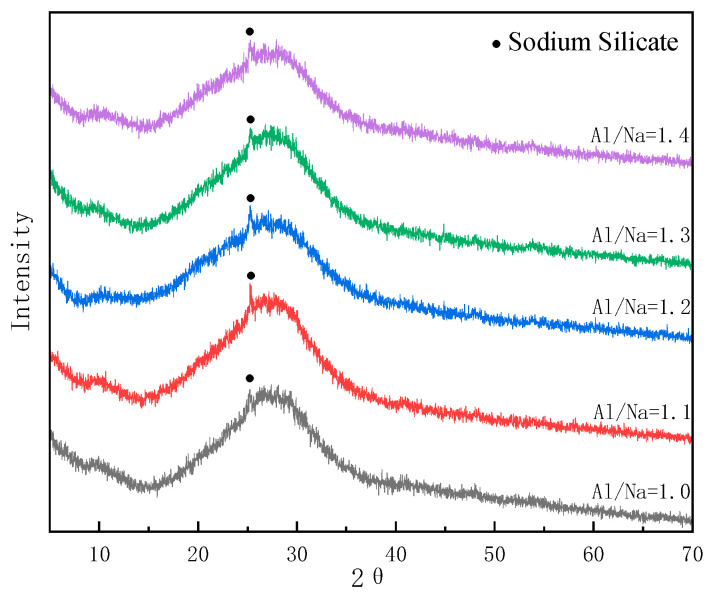
The XRD diagram of specimens with different Al/Na ratios (Si/Na = 2.0; age = 28 days).

The MIP (mercury intrusion porosimetry) method was used to test the pore structures of the geopolymer specimens. The porosity, most probable pore size and pore size distribution for different Si/Na ratios are shown in Figure 11. The most probable pore size first decreased and then slightly increased with the increase in Si/Na ratio. When the Si/Na ratio was 2.0, the most probable pore size reached its minimum value. Due to the special properties of the SiO4 tetrahedrons, they needed two metal cations to balance the negative charge of the systems. More SiO4 tetrahedrons were formed when the Si/Na ratio exceeded 2.0. However, the amount of Na^+^ metal cations was insufficient, so the most likely pore size hardly changed. It can also be seen from Figure 12 that the porosity of the geopolymers first increased and then decreased as the Si/Na ratio increased because the systems contained more Na^+^ when the Si/Na ratios were relatively small. Meanwhile, the base activator had a stronger alkalinity, which promoted geopolymerization. The gels formed in the early stages quickly bonded to the unreacted raw material particles and hardened too quickly, which led to the inability of the geopolymers to form dense structures. The alkalinity of the activator became weaker with the increase in Si/Na ratio but the active silicon contents in the systems increased, which promoted geopolymerization and made the gels form denser structures.

### 3.5. Phase Composition Analysis

#### 3.5.1. Changes in Phase Composition with Changes in Al/Na Ratio

The phase components of the sub-kaolin-based polymers with different Al/Na ratios after 28 days of curing are shown in Figure 12. The metakaolin formed the material composition of the slurry after hydration and there was a characteristic peak distribution in the range of 2θ = 20–35°. This indicated that the N-A-S-H gel, the main hydration product of the raw material, existed in an amorphous form. Some unreacted sodium silicate crystal phase (sodium silicate) was also detected in the sample powders. It can be seen from the figure that with the gradual increase in Al/Na ratio, the characteristic peak shapes of the sample powders at 2θ = 20–35° showed the trend of first strengthening and then weakening. As a result, the hydration reaction levels of the raw materials first increased and then decreased with the increase in Al/Na ratio.

#### 3.5.2. Changes in Phase Composition with Changes in Si/Na Ratio

**Figure 13:**
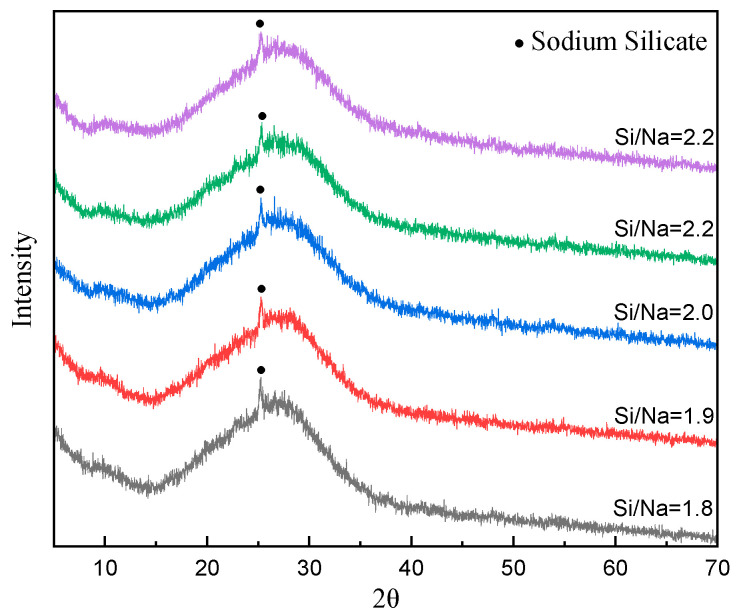
The XRD diagram of specimens with different Si/Na ratios (Al/Na = 1.3; age = 28 days).

The phase components of the sub-kaolin-based polymers with different Si/Na ratios after 28 days of curing are shown in Figure 13. Since all of the raw materials used in this experiment were metakaolin, the changes in Al/Na and Si/Na ratios mainly affected the reaction levels. In contrast, the phase components of the metakaolin-based geopolymers were basically the same for different Al/Na and Si/Na ratios. The main hydration product (N-A-S-H gel) existed in an amorphous form and there was some incompletely reacted sodium silicate crystal phase (sodium silicate). It can be seen from the figure that the characteristic peak shapes of the sample powders at 2θ = 20–35° became weaker as the Si/Na ratio increased. This showed that the hydration reaction levels of the raw materials tended to decrease with the increase in Si/Na ratio.

## 4. Conclusions

- With an increase in the Al/Na ratio, the Al contents of the systems increased and more OH^−^ was consumed to generate AlO_4_ tetrahedrons, so the residual OH^−^ contents after the reactions decreased and the pH of the pore solutions continued to decrease.
- With an increase in the Si/Na ratio, the alkalinity of the initiator became weaker, but the amount of excipient increased and the total OH^−^ contents in the systems remained almost unchanged. The OH^−^ concentrations on the surfaces of the raw material particles decreased, resulting in lower reaction levels and less OH^−^ being consumed. Therefore, the residual OH^−^ contents after the reactions increased and the pH of the pore solutions increased.
- The compressive strength of the geopolymers first increased and then decreased with an increase in the Al/Na ratio. The highly active Al increased the hydration reaction levels, which made the geopolymer structures become denser and demonstrate higher compressive strength. The Al components in the systems were excessive and the alkali contents gradually decreased when the Al/Na ratio was too high, which meant that the reaction levels and compressive strength of the geopolymers decreased.
- The compressive strength of the geopolymers gradually decreased with an increase in the Si/Na ratio. An increase in the Si/Na ratio led to a decrease in the alkali contents in the systems and the amount of metakaolin. Then, the reaction rates and levels of geopolymers decreased, so the compressive strength of the geopolymers also decreased.
- The exothermic reaction rates and the cumulative exothermic reactions of the geopolymers first increased and then gradually slowed down with an increase in the Al/Na ratio. This showed that an increase in the Al/Na ratio made the reaction levels first increase and then decrease. The exothermic reaction rates and the cumulative exothermic reaction of the geopolymers gradually decreased with an increase in the Si/Na ratio, which indicated that an increase in the Si/Na ratio made the reaction levels decrease.
- The SEM and MIP results showed that the smaller the pH value of a geopolymer pore solution, the denser the microstructure. Thus, the microstructures had good correlations with the changes in the pH values of the geopolymer pore solutions. The phase compositions after the hydration reactions were basically the same since the Al/Na and Si/Na ratios mainly affected the reaction levels of the geopolymers.

This study mainly focused on metakaolin-based geopolymers, but most of the composite gel systems were made from a variety of raw materials and the hydration processes of the composite systems were more complicated, so the pH values of the pore solutions from the composite-gelling systems could also be studied. In addition, in engineering practice, the decrease in the alkalinity of cement concrete due to CO_2_ invasion is one of the causes of steel corrosion, so changes in the pH values of pore solutions after the carbonization of geopolymers should be further investigated.

## Figures and Tables

**Table 1:** The chemical components of metakaolin (mass fractions).

Raw Material	SiO_2_	Al_2_O_3_	Fe_2_O_3_	MgO	Na_2_O	K_2_O	Others
Amount (%)	54.5	43	1.0	0.8	0.1	0.4	0.2

**Table 2:** The mix ratios of the metakaolin-based geopolymers that were tested in this study.

Sample	Al/Na	Si/Na	Sodium Silicate Dosage (g)	NaOH Dosage (g)	Amount of Deionized Water (g)
A1	1.0	2.0	175.89	14.74	26.83
A2	1.1		141.23	15.42	28.19
A3	1.2		112.36	15.98	29.32
A4	1.3		87.93	16.45	30.28
A5	1.4		66.93	16.86	31.10
S1	1.3	1.8	58.61	19.62	36.25
S2		1.9	73.27	18.03	33.26
S3		2.0	87.93	16.45	30.28
S4		2.1	102.59	14.87	27.30
S5		2.2	117.25	13.29	24.31

## Data Availability

Data are contained within the article.

## References

[1] Davidovits J. (1991). Geopolymers: Inorganic polymeric new materials. J. Therm. Anal. Calorim..

[2] Awoyera P.O., Adesina A., Sivakrishna A., Gobinath R., Kumar K.R., Srinivas A. (2020). Alkali activated binders: Challenges and opportunities. Mater. Today Proc..

[3] Peiliang C., Yaqian C. (2021). Advances in geopolymer materials: A comprehensive review. J. Traffic Transp. Eng. Engl. Ed..

[4] Komnitsas K., Zaharaki D. (2007). Geopolymerisation: A review and prospects for the minerals industry. Miner. Eng..

[5] Ahmaruzzaman M. (2010). A review on the utilization of fly ash. Prog. Energy Combust. Sci..

[6] Zhang B., Zhu H., Cheng Y., Huseien G.F., Shah K.W. (2022). Shrinkage mechanisms and shrinkage-mitigating strategies of alkali-activated slag composites: A critical review. Constr. Build. Mater..

[7] Ding Y., Dai J.-G., Shi C.-J. (2016). Mechanical properties of alkali-activated concrete: A state-of-the-art review. Constr. Build. Mater..

[8] Gao X., Yu Q.L., Brouwers H.J.H. (2015). Reaction kinetics, gel character and strength of ambient temperature cured alkali activated slag–fly ash blends. Constr. Build. Mater..

[9] Abdelli K., Tahlaiti M., Belarbi R., Oudjit M.N. (2017). Influence of the origin of metakaolin on pozzolanic reactivity of mortars. Energy Procedia.

[10] Imtiaz L., Rehman S., Memon S.A., Khan M.K., Javed M.F. (2020). A Review of Recent Developments and Advances in Eco-Friendly Geopolymer Concrete. Appl. Sci..

[11] Yang T., Zhu H., Zhang Z. (2017). Influence of fly ash on the pore structure and shrinkage characteristics of metakaolin-based geopolymer pastes and mortars. Constr. Build. Mater..

[12] Hadi M.N., Al-Azzawi M., Yu T. (2018). Effects of fly ash characteristics and alkaline activator components on compressive strength of fly ash-based geopolymer mortar. Constr. Build. Mater..

[13] Gharzouni A., Joussein E., Samet B., Baklouti S., Rossignol S. (2015). Effect of the reactivity of alkaline solution and metakaolin on geopolymer formation. J. Non-Cryst. Solids.

[14] Muñiz-Villarreal M.S., Manzano-Ramírez A., Sampieri-Bulbarela S., Gasca-Tirado J.R., Reyes-Araiza J.L., Rubio-Ávalos J.C., Amigó-Borrás V. (2011). The effect of temperature on the geopolymerization process of a metakaolin-based geopolymer. Mater. Lett..

[15] Rovnaník P. (2010). Effect of curing temperature on the development of hard structure of metakaolin-based geopolymer. Constr. Build. Mater..

[16] Tang S., Yao Y., Andrade C., Li Z. (2015). Recent durability studies on concrete structure. Cem. Concr. Res..

[17] Stefanoni M., Angst U., Elsener B. (2018). Corrosion rate of carbon steel in carbonated concrete—A critical review. Cem. Concr. Res..

[18] Lizcano M., Gonzalez A., Basu S., Lozano K., Radovic M. (2012). Effects of Water Content and Chemical Composition on Structural Properties of Alkaline Activated Metakaolin-Based Geopolymers. J. Am. Ceram. Soc..

[19] Shi Z., Lothenbach B. (2019). The role of calcium on the formation of alkali-silica reaction products. Cem. Concr. Res..

[20] Bernal S.A. (2015). The resistance of alkali-activated cement-based binders to carbonation. Handbook of Alkali-Activated Cements, Mortars and Concretes.

[21] Pouhet R., Cyr M. (2016). Carbonation in the pore solution of metakaolin-based geopolymer. Cem. Concr. Res..

[22] Su M., Zhong Q., Peng H. (2021). Regularized multivariate polynomial regression analysis of the compressive strength of slag-metakaolin geopolymer pastes based on experimental data. Constr. Build. Mater..

[23] Shi Z., Shi C., Wan S., Ou Z. (2017). Effect of alkali dosage on alkali-silica reaction in sodium hydroxide activated slag mortars. Constr. Build. Mater..

[24] Lv Y., Wang S., Ge Y., Xiao B., Peng H. (2021). CInfluence of slag mixing on alkalinity and geological polymerization behavior of polymer pore solution in metakaolin base. J. Cent. South Univ. Sci. Technol..

[25] Wang H., Wu H., Xing Z., Wang R., Dai S. (2021). The Effect of Various Si/Al, Na/Al Molar Ratios and Free Water on Micromorphology and Macro-Strength of Metakaolin-Based Geopolymer. Materials.

[26] Lahoti M., Narang P., Tan K.H., Yang E.H. (2017). Mix design factors and strength prediction of metakaolin-based geopolymer. Ceram. Int..

[27] Wan Q., Rao F., Song S., García R.E., Estrella R.M., Patiño C.L., Zhang Y. (2017). Geopolymerization reaction, microstructure and simulation of metakaolin-based geopolymers at extended Si/Al ratios. Cem. Concr. Compos..

[28] Gao B., Jang S., Son H., Lee H.J., Lee H.J., Yang J.J., Bae C.J. (2020). Study on mechanical properties of kaolin-based geopolymer with various Si/Al ratio and aging time. J. Korean Ceram. Soc..

[29] Castillo H., Collado H., Droguett T., Sánchez S., Vesely M., Garrido P., Palma S. (2021). Factors Affecting the Compressive Strength of Geopolymers: A Review. Minerals.

[30] Noorpour M., Tarighat A. (2021). Effects of Si/Al ratio on structure, modulus of elasticity, and density in NASH geopolymer: A molecular dynamics simulation based on novel macromolecular model. J. Mol. Model..

[31] Berry E., Hemmings R., Cornelius B. (1990). Mechanisms of hydration reactions in high volume fly ash pastes and mortars. Cem. Concr. Compos..

[32] Newton C., Sykes J. (1987). The effect of salt additions on the alkalinity of Ca(OH)_2_ solutions. Cem. Concr. Res..

[33] Bérubé M.A., Frenette J., Rivest M., Vézina D. (2002). Measurement of the alkali content of concrete using hot-water extraction. Cem. Concr. Aggreg..

[34] Duchesne J., Berube M.A. (1994). Available alkalies from supplementary cementing materials. Mater. J..

[35] Figueira R., Sousa R., Coelho L., Azenha M., de Almeida J., Jorge P., da Silva C.J.R. (2019). Alkali-silica reaction in concrete: Mechanisms, mitigation and test methods. Constr. Build. Mater..

[36] Haque M.N., Kayyali O.A. (1993). Aspects of chloride ion determination in concrete. Mater. J..

[37] Paudel S.R., Yang M., Gao Z. (2020). pH Level of Pore Solution in Alkali-Activated Fly-Ash Geopolymer Concrete and Its Effect on ASR of Aggregates with Different Silicate Contents. J. Mater. Civ. Eng..

[38] Natkunarajah K., Masilamani K., Maheswaran S., Lothenbach B., Amarasinghe D., Attygalle D. (2022). Analysis of the trend of pH changes of concrete pore solution during the hydration by various analytical methods. Cem. Concr. Res..

